# Uncovering the spatio-temporal patterns and drivers of 30 years of greening in South UK landscapes

**DOI:** 10.1007/s10980-026-02383-2

**Published:** 2026-05-25

**Authors:** Guilherme Castro, Julia Koricheva, Justin Moat, Phil Wilkes, Tim Wilkinson, Cristina Garcia

**Affiliations:** 1 Department of Biological Sciences, Royal Holloway University of London, Egham, Surrey TW20 0EX UK; 2 Royal Botanic Gardens, Kew, Richmond, TW9 3AE UK

**Keywords:** Ecosystem functioning, Ecological timeseries, Greening, Land cover changes, NDVI, Spatial heterogeneity

## Abstract

**Context:**

Anthropogenic pressures are pushing ecosystems towards collapse by disrupting their structure and functioning. Whilst ecological responses to climate and land cover changes are widely addressed at the global scale, a notable knowledge gap remains at the landscape level—a critical scale for translating science into action**.**

**Objectives:**

To evaluate the impacts of anthropogenic pressures on ecosystem functioning at the landscape scale. Specifically, we assess the influence of climate trends and land cover changes on the spatial and temporal patterns of Normalized Difference Vegetation Index (NDVI) over a 30-year period (1995–2024).

**Methods:**

Using Sussex (UK) as a study area, we examined spatio-temporal patterns in climate trends and NDVI – used as a proxy for vegetation distribution and productivity—over three decades based on satellite imagery. We characterized the spatial heterogeneity of observed changes and evaluated the role of climate and land cover changes as drivers of long-term NDVI dynamics.

**Results:**

We found an overall increase in NDVI across the study area and all land cover types (MK *tau* value = 0.605, p-value < 0.01, TS slope = 0.0044 ∆NDVI yr⁻^1^), marginally correlated with increasing average temperatures (ρ ≈ 0.34, p = 0.067). This increase in NDVI showed spatially heterogenous patterns with distinct hotspots of NDVI change (Local Moran’s I) linked to changes in land cover types. We detected rising temperatures in Sussex (annual average temperatures: Mann–Kendall (MK) *tau* value = 0.297, p-value = 0.022, Theil-Sen (TS) slope = 0.026 ℃ yr⁻^1^), but rainfall levels have not changed significantly (annual average rainfall: MK *tau* value = 0.21, p-value = 0.108, TS slope = 4.91 mm yr⁻^1^).

**Discussion:**

These findings show a positive relationship between rising temperatures and vegetation greening, as reflected by NDVI gains with spatially heterogenous patterns associated with land cover changes and hotspots of NDVI change. Our study provides spatially explicit evidence to support effective landscape management strategies and inform policies by demonstrating the interaction between anthropogenic pressures at the landscape level. Because the landscape is a relevant scale at which environmental change and its outcomes are perceived, our results offer insights to address the effects of global and local changes.

**Supplementary Information:**

The online version contains supplementary material available at https://doi.org/10.1007/s10980-026-02383-2.

## Introduction

Climate change and land use and land cover changes (LULCC) stand out as two main drivers of anthropogenic changes with long-lasting effects on ecosystems’ structure and functions. (IPCC 2023). On the one hand, climate change can alter patterns of primary productivity by modifying temperature and rainfall patterns, as well as increasing the frequency of extreme weather events (*e.g.*, droughts and heatwaves). Depending on spatial and temporal contexts, this may lead to the depletion of carbon stocks and reduced water availability, which in turn may increase vegetation mortality and exacerbate the risk of wildfires (Ciais et al. 2005; Reichstein et al. 2013; Williams et al. 2013; Li et al. 2021; Wolf and Paul-Limoges 2023; Yin et al. 2023). On the other hand, LULCC simplify, fragment, and modify vegetation communities and their phenological patterns over time, potentially altering critical ecosystem functions, such as primary productivity (Erb et al. 2016; Afuye et al. 2024). All these pressures and their interacting effects on ecosystem functioning raise concerns about ecosystems’ capacity to adapt to future climate scenarios (Bergstrom et al. 2021; Blake et al. 2024; Johnson et al. 2024). In response to these challenges, international efforts to protect and restore ecosystems are gaining momentum. A prominent example is the Kunming-Montreal Global Biodiversity Framework (GBF), which calls for evidence-based restoration and monitoring of all ecosystems to ensure their integrity, connectivity and resilience by 2050 (Convention of Biological Diversity 2022). Achieving these ambitious targets requires reliable monitoring efforts to evaluate current trends, enhance predictive capabilities, and guide management strategies at both national and global levels. Recognised monitoring programmes exist at local scales, ranging from sampling plots of ca. 100 m to sites covering up to *ca*. 500 hectares (Savill et al. 2010; Markham et al. 2022; Pettorelli et al. 2025) and at global scales (worldwide coverage; Balvanera et al. 2022), while ecological monitoring at the landscape and regional levels (areas ranging from ca. 10 to 100 km; Forman 1995; Frazier 2024) remain largely underexplored. This represents a critical data and knowledge gap, as restoration and conservation efforts are often implemented at the landscape scale—where principles and patterns derived from local or global analyses may not hold (Gilby et al. 2018; Lindenmayer et al. 2022; Mitchell et al. 2024). Moreover, the interactions among drivers of global change and their effects on ecosystem functioning remain poorly understood, particularly at scales that have not been sufficiently examined, namely at the landscape-level (Pettorelli et al. 2025). These limitations constrain our ability to link ecological changes at the landscape scale (*e.g.,* LULCC) with overarching global drivers, such as climate change. Bridging the divide between local expressions of global changes and the landscape scale—where ecosystem services underpinning human wellbeing are generated—is therefore fundamental to developing climate adaptive strategies (*e.g.* in land use frameworks) and ecological restoration programmes (*e.g.* in local nature recovery strategies; Schulze et al. 1989; Wu 2013; Frazier 2024).

To address these knowledge and data gaps, remote sensing is acknowledged as a key technology for assessing and monitoring environmental change at high spatial and temporal resolutions (Pettorelli et al. 2014). Satellites equipped with different sensor technologies have been instrumental in advancing our understanding of ecological processes at various scales by providing consistent and cost-effective means to evaluate ecosystem’s structure and functioning. As an example, vegetation indices generated from the reflectance measured by satellite sensors can be linked to specific ecological processes (*e.g.* Normalized Difference Vegetation Index (NDVI) as a proxy for Net Primary Productivity (NPP) (Pettorelli et al. 2005;Pettorelli 2013), photosynthetic activity (Glenn et al. 2008; Chen et al. 2014), plant growth (González-Alonso et al. 2006; Vicente-Serrano et al. 2016), CO_2_ flux (Box et al. 1989; Welp et al. 2016), plant health (Mehmood et al. 2024), among others), therefore providing ecological insights across the globe (Pettorelli et al. 2014, 2005;Huang et al. 2021). Moreover, long-term satellite missions, such as Landsat and Terra/Aqua satellite programs, have recorded data for many decades, enabling us to study ecosystem changes over long time periods (Wulder et al. 2019). These timeseries analyses have proven helpful in identifying ecological trends, quantifying how ecosystems respond to environmental changes, detecting early warning signs of ecological change, identifying tipping points, and documenting vegetation shifts associated with LULCC, and the spread of pests and diseases (Coops et al. 2010; Shi et al. 2017; Liu et al. 2019; Smith et al. 2022; Seo and Kim 2024). Such long-term analyses are essential for gaining a better understanding of ecosystem stability, and for predicting future responses at various scales, including the landscape scale where published evidence is still scarce (Redhead et al. 2020; Van Meerbeek et al. 2021; Smith et al. 2022). With increasing data availability and growing computing resources, there is an expanding potential for remote sensing to monitor climate and ecosystem changes (*e.g.* through Essential Climate Variables (ECV) and Essential Biodiversity Variables (EBV); Ballari et al. 2023; Skidmore et al. 2021) and to support adaptive management and restoration efforts at the landscape-level (Foody 2023).

Here, we characterise the spatio-temporal patterns of NDVI over a 30-year period (1995 to 2024) across the county of Sussex (Southeast England) to evaluate the impact of climate and land cover changes on NDVI at the landscape scale. We focus on landscapes in the South of the UK as this region is projected to experience significant impacts under current and future climate change scenarios, including increased frequency of extreme high temperatures (Hanlon et al. 2021). Moreover, Sussex illustrates a landscape shaped by centuries of sustained management practises (Bannister 2010), while also experiencing processes of land use intensification. Nonetheless, a number of conservation and restoration actions have been implemented during the last decades, including nature-friendly farming systems, establishment of protected areas, and rewilding approaches (Schulte to Bühne et al. 2022). By combining spatial and temporal trends of climate and NDVI across the county of Sussex, we first investigate whether climate variables (annual temperatures and annual rainfall) exhibit significant changes over the 30-year period. As long-term trends previously published for the whole UK indicate increased temperatures and rainfall (Yule et al. 2023; Kendon et al. 2025), we also expect significant increases in annual temperatures (average, maximum and minimum) and in annual average rainfall levels. Secondly, we assess whether observed climatic trends correlate with NDVI temporal changes using NDVI as a proxy for vegetation distribution and productivity. Some authors have shown that NDVI significantly increased under current climate change trends (Li et al. 2025; Lu et al. 2025; Zhang et al. 2025), and if these trends are verified for our study area, we expect an overall increase in NDVI. Finally, we evaluate spatial heterogeneity in NDVI change patterns and test whether land cover changes (LCC) have influenced them. Given that different land uses affect vegetation composition and growth in distinct ways, we anticipate contrasting long-term trends and spatially heterogeneous patterns in NDVI change, with LCC significantly enhancing and/or hindering the greening of the landscape.

## Methods

### Study area

**Fig. 1:**
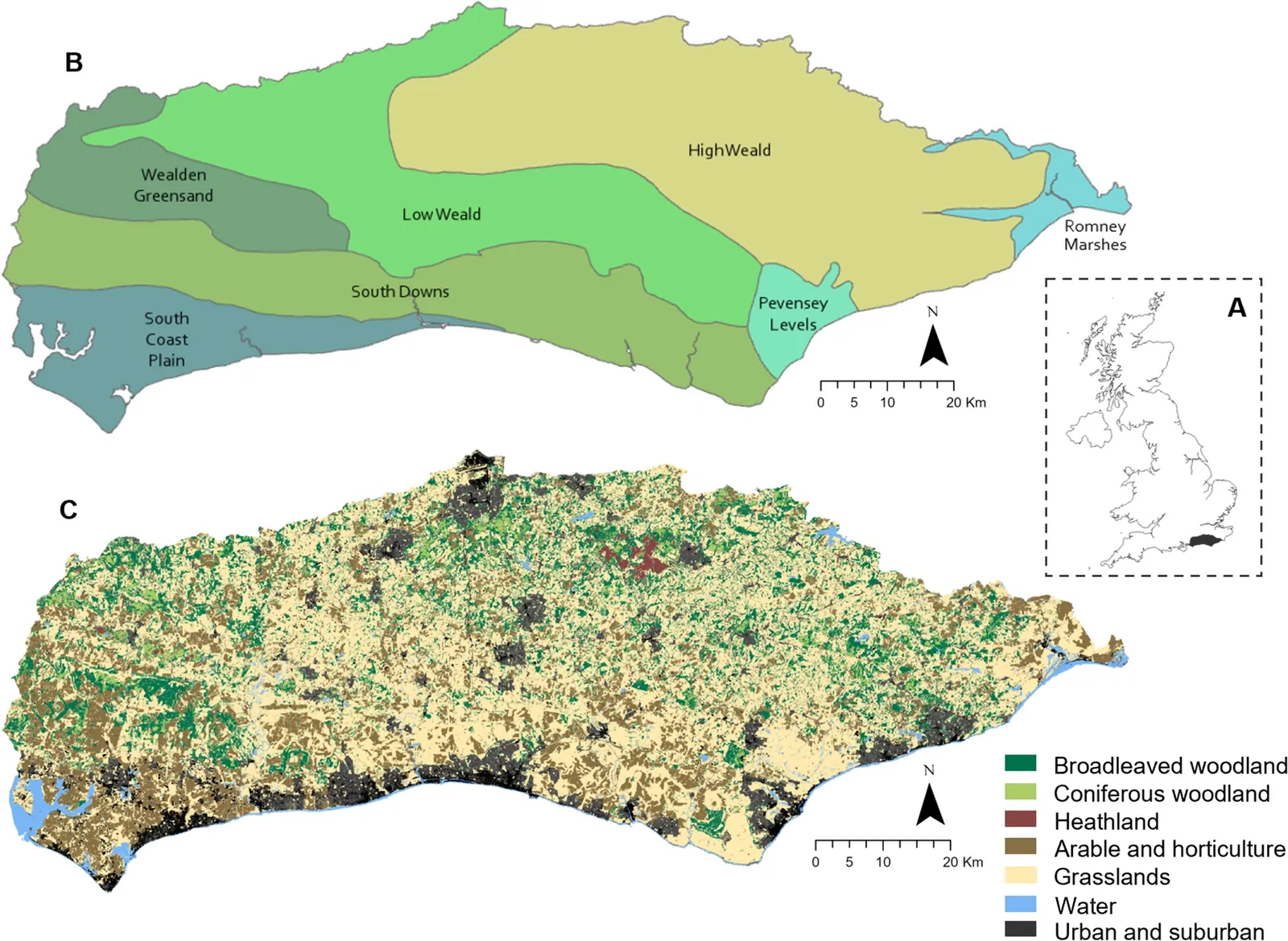
Map of the study area – Sussex, United Kingdom (UK). **A**—the location of the Sussex region (in dark) in relation to the UK; **B**—The spatial distribution of the different National Character Areas found in Sussex (Natural England 2024), and **C** – the 2023 land cover map of the study area based upon Land Cover Map 2023 © UKCEH 2024. Contains Ordnance Survey data © Crown Copyright 2007, Licence number 100017572 (Morton et al. 2024b)

Our study encompasses the county of Sussex (Southeast England; Fig. 1). The study region has a temperate climate with maritime influence, marked by moderate rainfall, cool winters, and warm summers (Bannister 2010). Sussex spans approximately 383,000 hectares and features a diverse landscape, ranging from exposed hilltops (with a maximum altitude of 279.7 m above sea level) to enclosed valleys, interspersed by undulating fields and flat coastal plains. Soil types range from nutrient-poor heavy clay to alkaline chalk and acidic sandy soils. The variety and combinations of soils types across the region have historically influenced land use management (Bannister 2010). Many areas across Sussex still retain numerous landscape features from medieval times—irregular-shaped fields, small woodlands, heath commons, and narrow woodland strips between fields. The resulting landscape is characterised by seven distinct National Character Areas (NCA) (Fig. 1). Sussex is dominated by farmland, with grasslands and arable fields covering around 68% of the region, typically small-sized fields surrounded by hedgerows and woodland patches (Morton et al. 2024b). Additionally, wood pastures have been particularly prevalent in the region, although many of those sites have transitioned into woodland habitats or converted into grasslands and arable fields over the last decades. Nonetheless, Sussex is among the most wooded counties in England (around 15% of its area is covered by woodlands) and has the highest ancient woodland cover (Bannister 2010). Coastal habitats can be found in the region, along with flat marshlands and urban coastal areas. Sussex comprises many designated areas with different levels of protection (e.g. National Park, Sites of Special Scientific Interest (SSSI) and Local Nature Reserves). Overall, more than 60% of Sussex's land area has some form of statutory protection. However, many of those designated areas do not meet the IUCN protection requirements for conservation purposes, such as National Parks and National Landscapes (Cunningham et al. 2021; Starnes et al. 2021).

### Data processing and analysis

We integrated climate, spectral imagery, and land cover datasets to analyse the temporal variation in the annual average values of climate and of vegetation greenness. Given that these datasets are available at various spatial scales, each analysis used the most suitable spatial scales to secure reliable results.

#### Climate

To investigate annual climate trends in our study area, we used climate data spanning the 30-year period from 1995 to 2024 from the Met Office UK and Regional Climate Observations dataset, with a resolution of 1 × 1 km. Pixels corresponding to our study area were aggregated to annual averages (Met Office 2025). We selected the 30-year period in line with the criteria for defining climate normals (National Centers for Environmental Information 2015). Climate variables analysed included annual rainfall (mm), and average, maximum and minimum annual temperatures (°C).

#### Normalized difference vegetation index (NDVI)

Satellite imagery collected with passive sensors, such as multispectral sensors, measures reflected light from vegetation at large spatial scales, allowing the generation of different spectral indices used as proxies for ecosystem functions (Pettorelli et al. 2014). To investigate ecosystem functioning patterns over our study area, we analysed data from the Landsat constellation from 1995 to 2024. This time period was selected to overlap with the climate data timescale (Sect. "Climate"). Among various available satellite constellations, we selected Landsat imagery as it provides historical data for the timescale considered with suitable spatial resolution (30-m resolution per pixel) for our study area. To characterize the ecosystem functioning variation, we selected a widely used spectral index as a proxy for vegetation distribution, productivity and dynamics—the Normalized Difference Vegetation Index (NDVI) (Tucker 1979; Pettorelli 2013):

$$ {\text{NDVI = }}\frac{NIR - Red}{{NIR + Red}} $$

where *Red* stands for Red band $(0.66\upmu \mathrm{m})$ and *NIR* for Near-Infrared band $\left(0.86\upmu \mathrm{m}\right)$. NDVI is a vegetation greenness index that uses a ratio of reflected red and near-infrared light. NDVI values range from −1 to + 1, where positive values show green vegetation, which absorbs visible light but highly reflects near-infrared, while negative values indicate the absence of vegetation (Pettorelli et al. 2005). A description of spatio-temporal patterns of NDVI enable us to characterize trends in ecosystem functioning and link them with underlying drivers of change, such as LULCC (Pettorelli et al. 2014, 2005). We used Landsat Collection 2 8-day NDVI composites generated from Tier 1 Level 2 orthorectified scenes (Google Earth Engine 2023; U.S. Geological Survey 2020). The analysed scenes covered the Worldwide Reference System paths/rows 201/24 and 201/25. To generate a pixel-scale annual average NDVI composite, we calculated the average for each pixel from all scenes available for each respective year (Table 1).

**Table 1:** Total number of NDVI scenes available per year from 1995 to 2024 and for each year quartile

Year	Total number of scenes	1st Quartile (1st Jan – 31st Mar)	2nd Quartile (1st Apr – 30st Jun)	3rd Quartile (1st Jul – 30st Sep)	4th Quartile (1st Oct – 31st Dec)
1995	25	6	7	9	3
1996	24	4	7	9	4
1997	27	5	6	9	7
1998	25	7	4	8	6
1999	28	3	7	10	8
2000	34	5	9	12	8
2001	40	9	10	11	10
2002	32	7	10	8	7
2003	23	7	5	7	4
2004	27	6	8	8	5
2005	25	5	5	9	6
2006	26	3	8	9	6
2007	25	9	5	8	3
2008	13	1	7	4	1
2009	31	7	8	10	6
2010	24	5	8	7	4
2011	26	1	8	10	7
2012	15	1	3	5	6
2013	24	0	5	12	7
2014	40	12	10	9	9
2015	38	10	11	10	7
2016	40	11	10	9	10
2017	34	5	10	10	9
2018	31	5	9	11	6
2019	32	7	8	10	7
2020	34	8	10	11	5
2021	28	5	8	7	8
2022	40	9	11	10	10
2023	40	9	11	12	8
2024	35	7	10	10	8
Total	886	179	238	274	195

From the resulting yearly average composites, we calculated the annual average, standard deviation, maximum and minimum NDVI values. We applied the same spatial procedure to five land cover types in the study area which are the most representative land cover types (covering 84.1% of the whole study area), namely broadleaved woodlands, coniferous woodlands, grasslands, arable and heathland. We combined different habitat classes referring to the same land cover type (*e.g*. different types of grasslands) and excluded all the other land cover types from our target study area, such as urban areas and water bodies using the most recent land cover map (2023) (Morton et al. 2024b). Additionally, we filtered the 98th percentile NDVI values to avoid spectral artefacts in our analysis. We used Google Earth Engine (Gorelick et al. 2017) to download Landsat products, generate composites and calculate statistical parameters (annual average, maximum and minimum NDVI).

#### Statistical analysis

We applied a non-parametric Mann–Kendall (MK) analysis to test whether climatic variables and NDVI values followed a monotonic upward or downward trend over the whole study period. The MK test estimates a correlation *tau*’s parameter that ranges from -1 to 1, with positive values suggesting a monotonic positive trend, where observations recorded at time *i* tend to be higher than those at time *i-1*. Negative values show declining trends, and zero values (at p < 0.05) indicate that the observed values recorded at time *i* are independent of those at time *i-1*. MK values below 0.3 are considered a weak correlation, while between 0.3 and 0.7 a moderate correlation and above 0.7 a strong correlation (Akoglu 2018). We applied MK test to the overall study area and to each land cover type using the *Kendall* R package (McLeod 2022). Alongside MK tests, we performed non-parametric Theil-Sen slope tests to climate variables and annual average NDVI to assess rates of change and confidence intervals using *Trend* R package (Pohlert 2023). We also applied a non-parametric Spearman’s test to assess the correlation between annual average temperature and annual average NDVI using *cor.test* function in R 4.4.1 (R Core Team 2024).

To investigate the NDVI spatial distribution patterns over time we applied:

i) pixel-scale MK analysis using yearly average NDVI composites produced from Landsat Collection 2 8-day composites (U.S. Geological Survey 2024). To account for multiple comparisons across pixels in MK analysis, we controlled the False Discovery Rate (FDR) using Benjamini Hochberg (BH) method (q = 0.05). MK p-values were ranked and compared to the BH q-threshold. Pixels with p-values below this threshold were considered statistically significant. The corrected FDR significance results were then used to generate significant trend results. We calculated the proportion (%) of the total number of significant positive and negative MK *tau* values and applied a Chi-square test to determine whether the observed counts of statistically significant positive and negative MK *tau* values differ from an equal distribution (null hypothesis). We also tested the normality, skewness and kurtosis of all MK *tau* values. To map the pixel-scale spectral trends and apply the statistical tests, we used the ‘Scipy’ (Virtanen et al. 2020) and ‘Rasterio’ (Gillies 2013) Python libraries.
ii) spatial autocorrelation analysis to assess the spatial configuration patterns of MK *tau* values, *i.e*. if the trend spatial analysis showed randomly or clustered distribution patterns across the study area. We applied a Local Moran's I statistic test to the MK *tau* value raster to assess spatial autocorrelation patterns. Given the non-parametric and skewed distribution of MK *tau* values, we applied the *k* Nearest Neighbours method (499 permutations). To determine an appropriate k value, we evaluated Global Moran’s I across a range of neighbourhood sizes (from 2 to 100). As expected, Global Moran’s I decreased with increasing k, while z-scores increased monotonically. We selected *k* = 8 due to its high and significant Global Moran’s I value (0.842, p < 0.001), indicating strong spatial clustering of MK values (Appendix 1). This neighbourhood size balances the detection of significant local spatial autocorrelation while minimizing the risk of spatial smoothing that could obscure hotspots of NDVI change. Similarly to the pixel-scale MK analysis, we applied FDR correction (q = 0.05) to avoid false positives. We converted the MK *tau* value raster to a point layer as a input for the spatial autocorrelation analysis, which was performed using the Spatial Autocorrelation tool (Global Moran’s I) and the Cluster and Outlier Analysis tool (Anselin Local Moran's I) in ArcGIS Pro 3.2.1 (Environmental Systems Research Institute (ESRI), 2023).
iii) land cover change analysis from 1990 to 2023 to assess changes in land cover types. We used two available Land Cover Maps (LCM) at 25 m resolution obtained from UK Centre for Ecology & Hydrology (UKCEH Rowland et al. 2020; Morton et al. 2024a;) for our study area that better aligned with the years of 1995 and 2024. Although both datasets show a consistent land cover type classification schema, they differ in both sensor sources and classification methodologies, which may lead to some misclassifications and uncertainty. To both LCM, we applied a nearest neighbour resampling to 30 m resolution to match satellite imagery pixel resolution. We assessed any pixel-scale LCC from 1990 to 2023 by generating a binary land cover change map (0 – no land cover change; 1 – land cover change) and a transition matrix identifying land cover transitions for each pixel, producing all possible transitions among the five land cover types considered in the study. The number of pixels associated with each transition was calculated to quantify the extent of land cover stability and change across the study area. MK *tau* values were then extracted for each transition to characterize the MK *tau* distribution profiles. To test whether MK *tau* values differed between land cover transitions and the corresponding stable land cover type, *i.e.* where no LCC were detected, a Kruskal–Wallis test was applied to compare the distributions of MK *tau* values among transitions within each land cover type. When significant differences were detected, Dunn’s post-hoc test was used to identify transitions that differed significantly from the stable land cover type. We used Google Earth Engine (Gorelick et al. 2017) to generate the different transition rasters and used *terra, dplyr, FSA,* and *tidyr* R packages (Wickman et al. 2025; Derek Ogle 2026; Robert and Hijmans 2026; Wickham 2026) to apply the LCC transition analysis and the statistical tests.

## Results

### Climate temporal trends

**Fig. 2:**
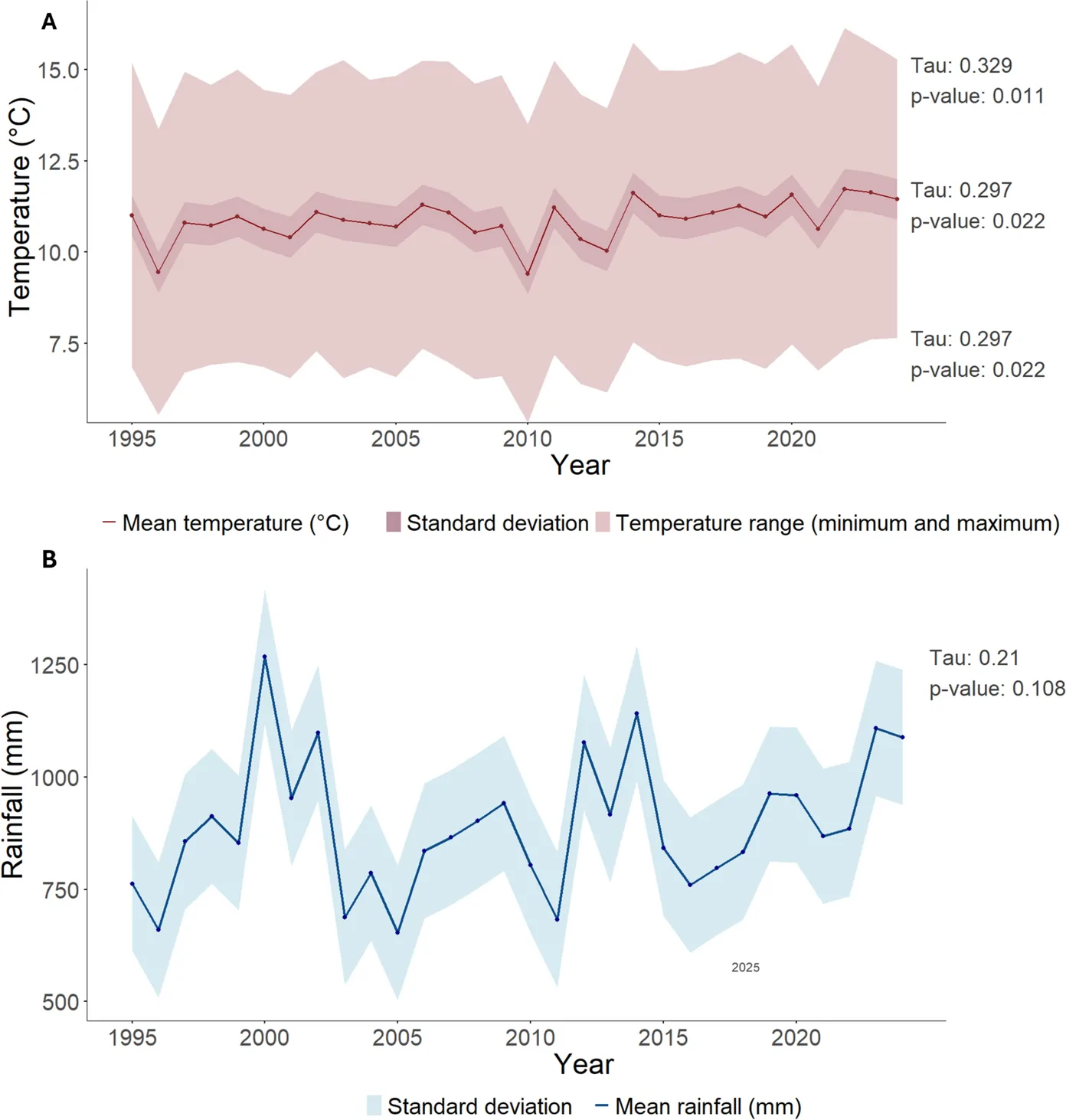
Climate trends from 1995 to 2024 (A – Temperature; B – Rainfall) in Sussex from 1995 to 2024. Mann–Kendall test *tau* values and their associated p-values are shown for average, maximum and minimum temperatures (**A**) and for rainfall (**B**), all N = 30

As hypothesised, our climate analysis showed a significant overall increase of average (MK *tau* value = 0.297**,** N = 30, p-value = 0.022, Theil-Sen slope = 0.0260 ℃ yr⁻^1^, CI 95% = [0.0057; 0.0432]), maximum (MK *tau* value = 0.329**,** N = 30, p-value = 0.011, Theil-Sen slope = 0.0290 ℃ yr⁻^1^, CI 95% = [0.0041; 0.0503]) and minimum temperatures (MK *tau* value = 0.297**,** N = 30, p-value = 0.022, Theil-Sen slope = 0.0209 ℃ yr⁻^1^, CI 95% = [0.0043; 0.0401]) from 1995 to 2024. For the average temperature, the cumulative increase was 0.78 ℃ during the study period. However, contrary to our expectations, rainfall data did not show any significant monotonic change for the same study period (MK *tau* value = 0.21**,** N = 30, p-value = 0.108, Theil-Sen slope = 4.91 mm yr⁻^1^, CI 95% = [− 1.08; 11.75]; Fig. 2).

### NDVI temporal trends

**Fig. 3:**
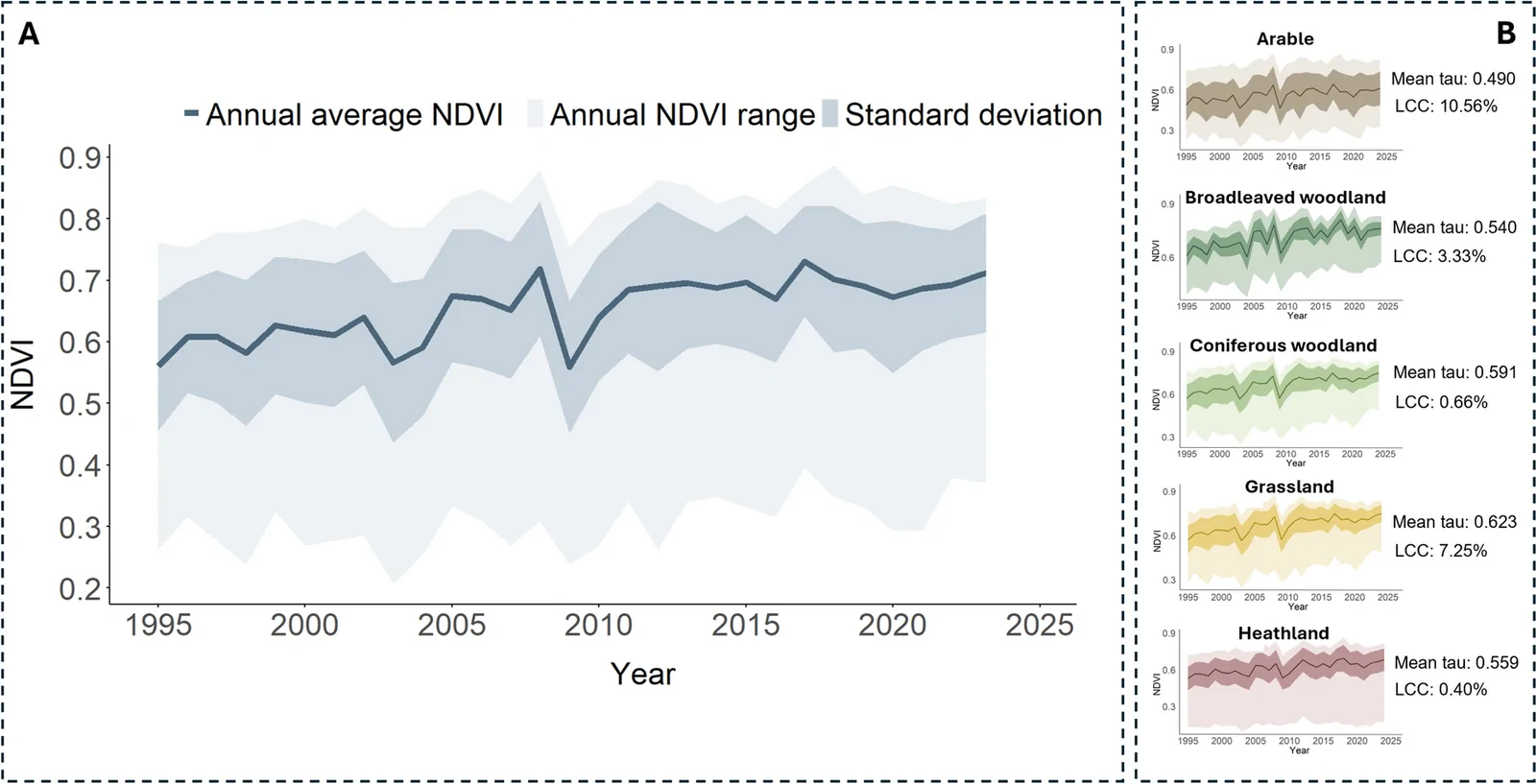
Temporal variation in NDVI values from 1995 to 2024 for the whole study area (**A**) and for each land cover type included in the study (**B**). In all graphs, the shadowed area around the annual average trend line shows the annual standard deviation, and the light shadow shows the range between annual minimum and annual maximum NDVI values. All average MK *tau* values have p-values < 0.01. LCC from 1990 to 2023, per land cover type, relative to the total study area, is shown to account for LCC in NDVI trends

In line with our expectations of increased NDVI values, we observed a significant moderate increase in the annual NDVI values: average (MK *tau* value = 0.605**,** N = 30, p-value < 0.01, Theil-Sen slope = 0.0044 ∆NDVI yr⁻^1^, CI 95% = [0.0030; 0.0056]), minimum (MK *tau* value = 0.425**,** N = 30, p-value < 0.01, Theil-Sen slope = 0.0028 ∆NDVI yr⁻^1^, CI 95% = [0.0019; 0.0039]), and maximum (MK *tau* value = 0.522**,** N = 30, p-value < 0.01, Theil-Sen slope = 0.0035 ∆NDVI yr⁻^1^, CI 95% = [0.0016; 0.0049]) for our study area. Additionally, all land cover types showed positive monotonic trends, with MK *tau* values ranging from *tau* = 0.49 in arable to *tau* = 0.623 in grasslands (all p < 0.01), suggesting that the greening effect occurs at different intensities across land cover types (Fig. 3).

A positive marginally significant correlation was observed between annual average NDVI and annual average (Spearman correlation, ρ ≈ 0.34, N = 30, p = 0.067) and annual minimum temperatures (Spearman correlation, ρ ≈ 0.35, N = 30, p = 0.055) but we have not detected any correlation between annual average NDVI and annual maximum temperatures (Spearman correlation, ρ ≈ 0.31, N = 30, p = 0.1).

### Spatio-temporal NDVI patterns

**Fig. 4:**
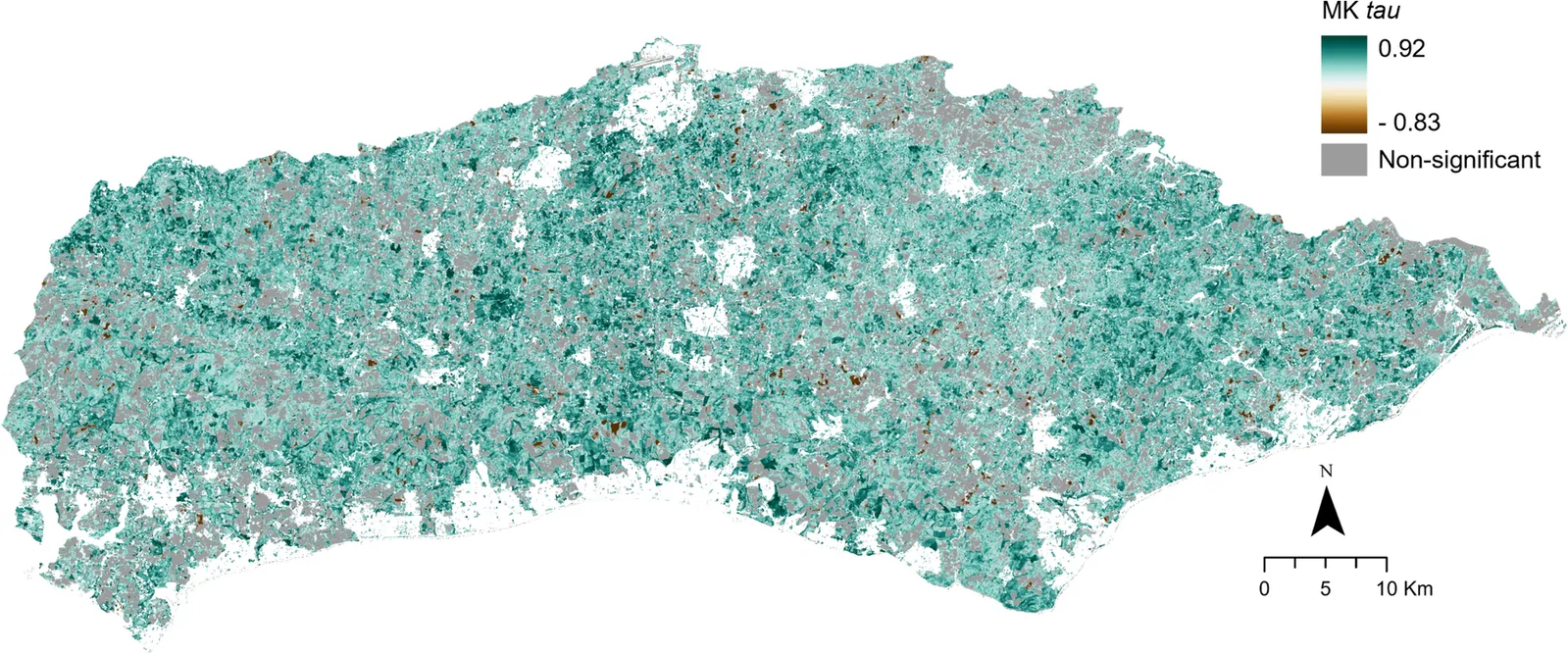
Map of the *tau* values after applying a pixel-scale Mann–Kendall NDVI trend analysis across the study area from 1995 to 2024. Pixels showing positive MK *tau* values show an increase in NDVI and negative MK *tau* values indicate a decrease in NDVI

NDVI values showed a significant monotonic positive trend, *i.e*. values tend to increase with time (MK test) in an area covering 68.2% of the study area. Among those pixels that showed a significant trend, 98.95% of pixels presented a positive trend (increasing NDVI values with time) (Fig. 4; Chi-square = 71,789.8923, p-value = 0.00). The pixel-scale MK map can be interactively visualised at: (https://guivcastro.projects.earthengine.app/view/greening).

**Fig. 5:**
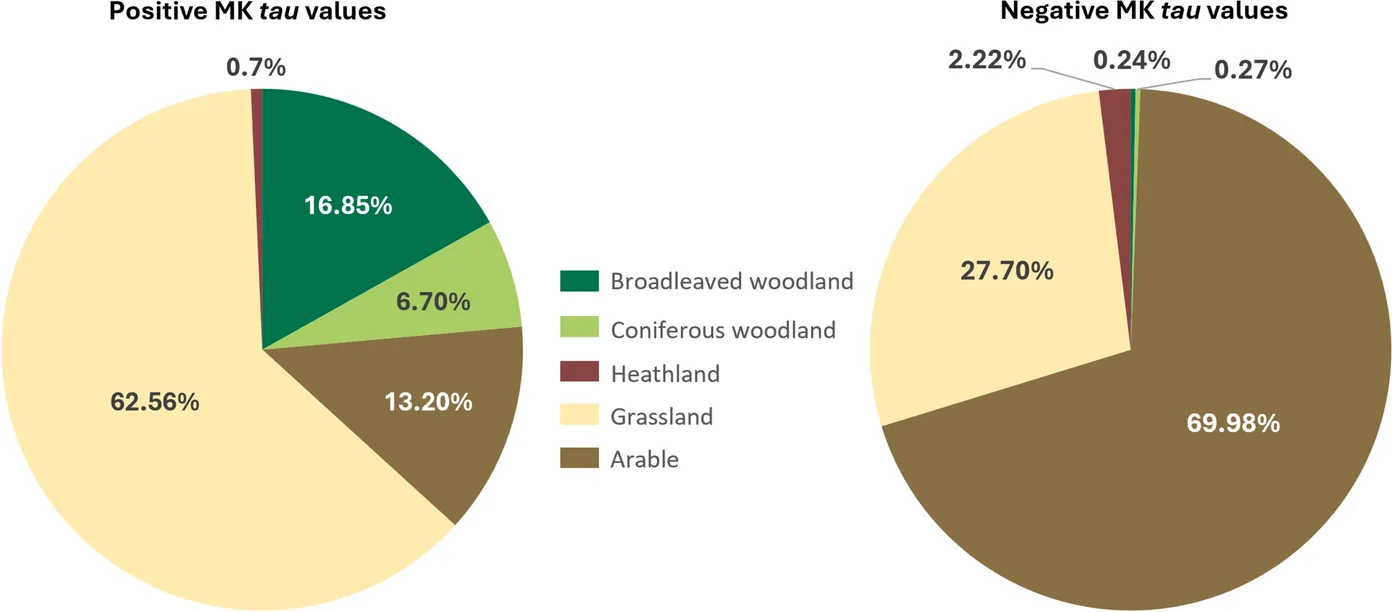
Pie charts showing the proportion of positive (left) and negative (right) Mann–Kendall *tau* values across the study area by land cover type

The distribution of MK values was left-skewed and showed a leptokurtic pattern, indicating the presence of both strong positive and negative MK values in the study area (Appendix 2). Our analyses also show that land cover types differed in the proportion of positive and negative MK values (Chi-square test = 71,789.8923; p-value = 0.00, Fig. 5). Grasslands had the highest proportion of positive values and heathland the lowest, whereas negative MK values were mainly observed across arable land and grasslands (Fig. 5).

**Fig. 6:**
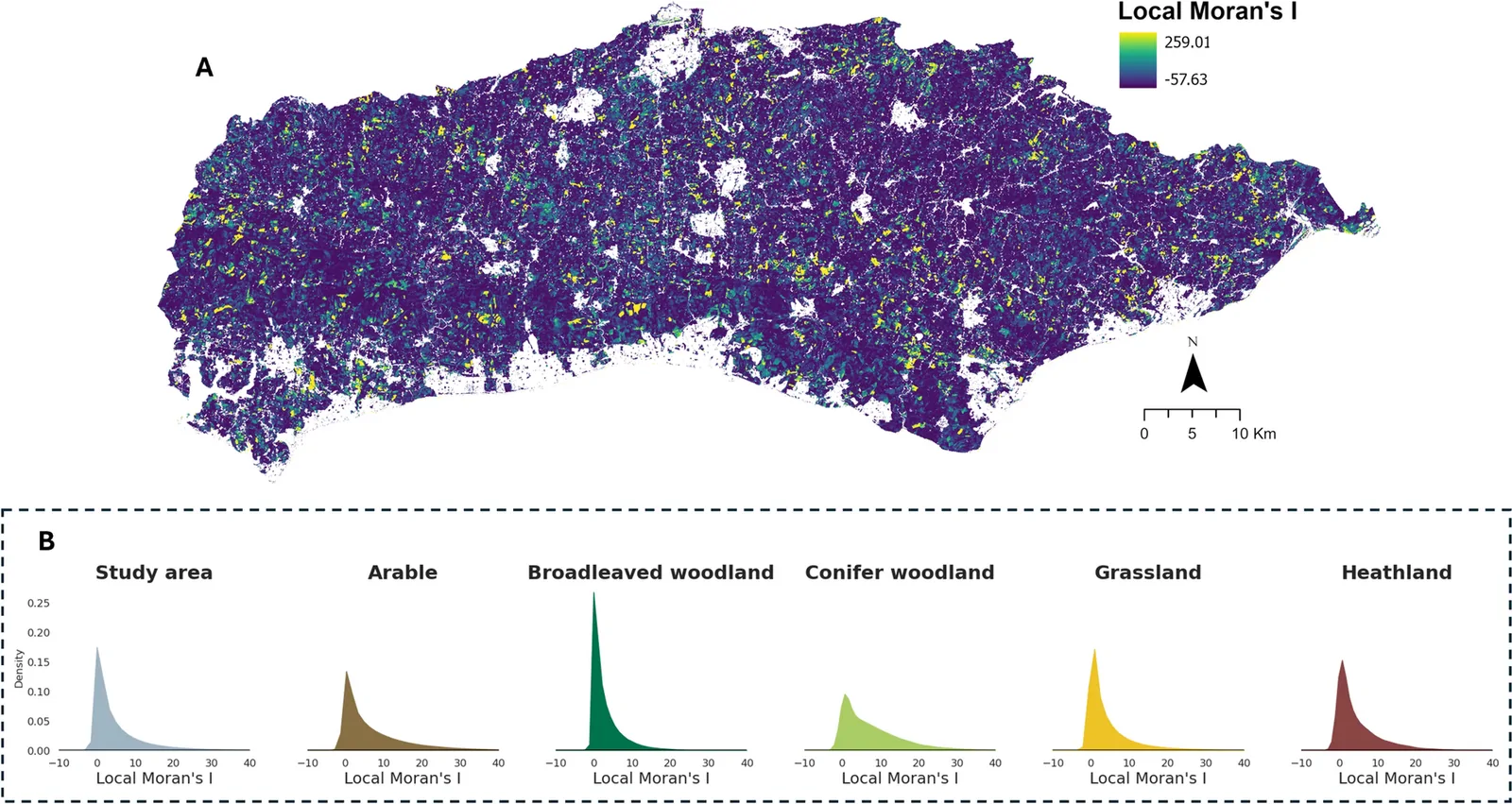
**A** Map of the spatial distribution of the local Moran’s I values estimated for MK *tau* values representing NDVI change. Pixels showing higher positive Local Moran’s I values represent pixels with clustered distributions (green to yellow), while pixels showing close to zero Local Moran’s I values represent pixels with random distribution (blue to purple). White pixels represent areas not included in the analysis (*e.g*. urban areas and water bodies). **B** – Graphs showing the distribution of Local Moran’s I values for the entire study area and for each land cover type. The x-axis range was restricted to exclude extreme outliers to enhance data visualization. These plots with the full x-axis range of values are provided in the *Supplementary materials* section (Appendix 3)

Spatial autocorrelation analysis showed that MK values were spatially arranged in both random and clustered distributions (Fig. 6). The Local Moran's I analysis revealed that 47.3% of pixels had significant spatial autocorrelation (p-value ≤ 0.05), thus representing areas where similar MK values tend to cluster, while 52.7% of the pixels show a random distribution of MK values (p-value > 0.05). These clusters of NDVI change represent areas that show either a strong positive (High-High) or strong negative (Low-Low) MK values, surrounded by areas with moderate increases or decreases in NDVI. The proportion of pixels aggregated in High-High clusters were slightly more frequent than the proportion of pixels aggregated in Low-Low clusters (56.3% *vs.* 42.9%). A smaller proportion of pixels showed positive values surrounded by low values (High-Low – 0.5%), as well as low values surrounded by high values (Low–High – 0.3%). For the study area and all land cover types, the Local Moran’s I values showed a right-skewed distribution with most points close to a random distribution (near 0) and a smaller proportion of points associated with clustered distributions (positive values). Additionally, the distribution of Local Moran’s I values between different land cover types shows that some land cover types present a higher proportion of clustered pixels (*e.g.* coniferous woodlands) when compared with other land cover types (*e.g.* broadleaved woodlands). This variation in the distribution of spatial autocorrelation values (random *vs.* clustering) between land cover types, along with the differing proportions of these values across the study area, highlights spatial heterogeneity in NDVI change over time. This supports our expectation that land-cover changes underpin changes in NDVI patterns.

### Land cover changes

**Fig. 7:**
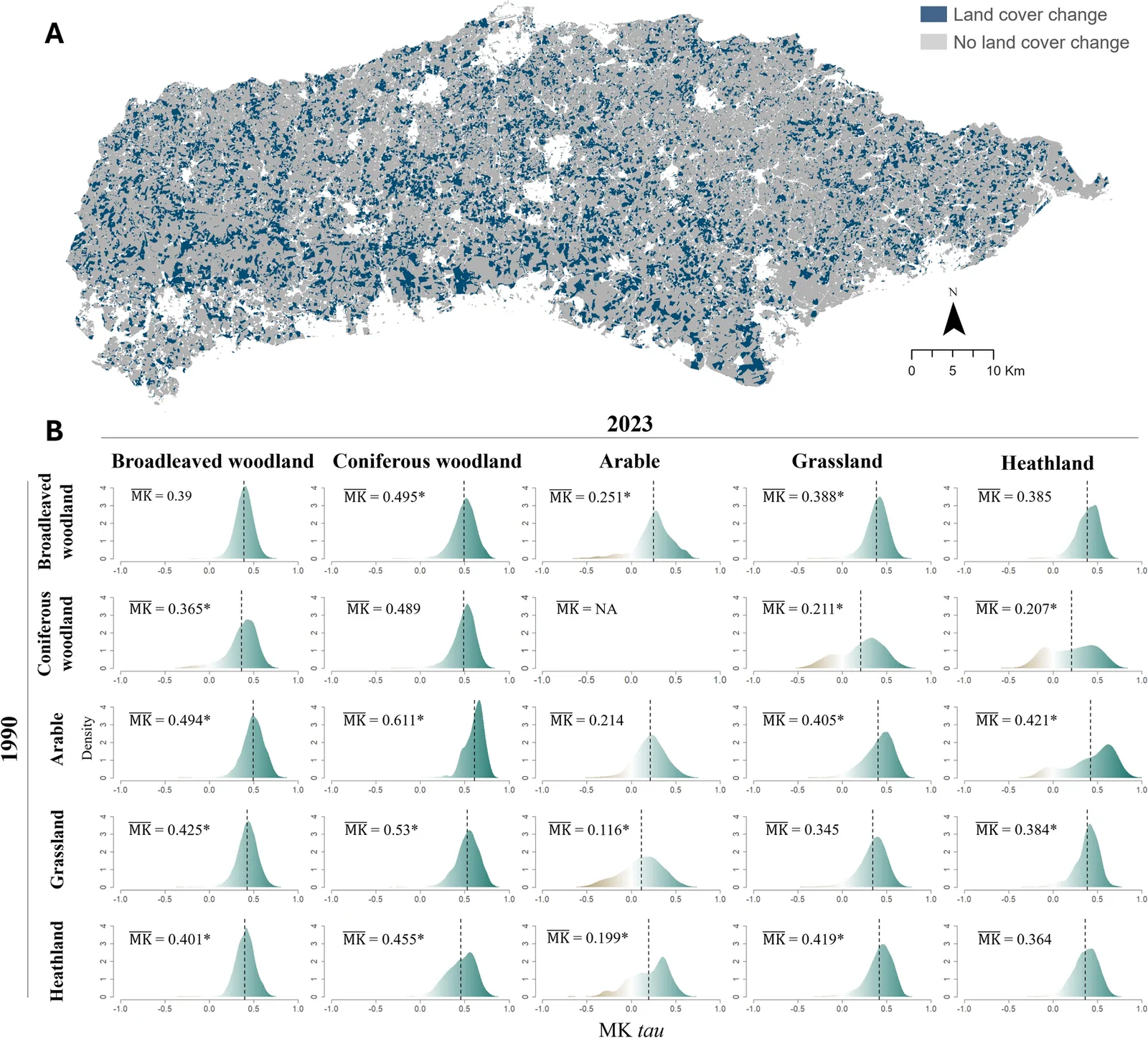
Land cover changes and effects on NDVI trends. **A** – Land cover change from 1990 to 2023; **B**—Transition matrix (from 1990 to 2023) of MK *tau* values according to land cover changes. $\overline{\mathrm{M}\mathrm{K} }$ represents the average MK *tau* for each land cover type distribution. $\overline{\mathrm{M}\mathrm{K} }$ values marked with * indicate significant difference compared to the no-change transition for the relevant land cover type

Our results show that land cover changes occurred in 22.43% of the pixels across the study area from 1990 to 2023 (Fig. 7). These changes occurred in varying directions and proportions across land cover types (Table 2). Coniferous woodlands and arable fields had the lowest and highest proportions of land cover changes, respectively.

**Table 2:** Transition matrix of land cover changes (LCC). The matrix shows: i) area (km^2^) for each land cover transition; ii) proportional (%) area of each transition per land cover type; iii) total LCC area (km^2^) per land cover type, and iv) proportion of LCC (%) for each land cover type

		2023						
		Broadleaved woodland (Km^2^)	Coniferouswoodland (Km^2^)	Arable (Km^2^)	Grassland (Km^2^)	Heathland (Km^2^)	Total LCC area (Km^2^) per land cover type	Proportion of LCC (%) per land cover type
1990	Broadleaved woodland	497.65 (82.68%)	38.97 (6.61%)	2.54 (0.43%)	59.02 (10.01%)	1.57 (0.27%)	109.38	14.98%
	Coniferous woodland	16.37 (24.36%)	45.65 (67.97%)	0.00 (0.0%)	3.99 (5.94%)	1.16 (1.73%)	21.85	2.99%
	Arable	6.08 (0.76%)	0.37 (0.05%)	458.23 (57.58%)	330.98 (41.60%)	0.07 (0.01%)	347.54	47.58%
	Grassland	47.86 (2.87%)	5.35 (0.32%)	176.65 (10.60%)	1,434.39 (86.11%)	1.63 (0.10%)	238.28	32.63%
	Heathland	3.88 (14.33%)	1.13 (4.18%)	1.90 (7.00%)	6.01 (22.20%)	14.16 (52.29%)	13.27	1.82%

Changes in land cover types indicate shifts in vegetation composition and structure. Therefore, we expected to find differing NDVI values in areas where such changes occurred (Fig. 7). Areas where no LCC were detected generally exhibited moderate positive trends, with average MK values ranging from 0.214 in arable fields to 0.489 in coniferous woodlands. Transitions to woodland habitats (both coniferous and broadleaved) showed strong greening trends, particularly from arable fields to coniferous woodlands (MK *tau* = 0.611). In contrast, transitions towards arable fields generally exhibited weaker NDVI trends, such as from grasslands to arable (MK *tau* = 0.116). Overall, all land cover types showed at least three LCC with significant differences in MK *tau* values in relation to their corresponding stable land cover types. These results indicate that LCC had distinct (*i.e.* both positive and negative) but consistent effects on NDVI trends across the landscape.

## Discussion

In this study, we used satellite imagery to reveal changes in NDVI from 1995 to 2024 and assessed how these changes were distributed across different land cover types in Sussex (UK). Additionally, we also identified the impacts of underlying drivers (climate and land cover changes). Our results showed that i) annual temperatures (average, maximum and minimum) – but not rainfall—have significantly increased over our study period; ii) NDVI has increased throughout the study area and for each land cover type considered, and these changes were marginally significantly correlated with changes in average and minimum temperatures; iii) NDVI showed a spatially heterogenous change during the study timeframe and, iv) LCC had a significant impact in NDVI trends.

The rising trend in average temperature aligns with national and global trends (Copernicus 2024; Kendon et al. 2024). Increasing temperatures are reported to have mixed effects on NDVI, with observed variations explained by different vegetation responses. Some studies suggest that rising temperatures may cause higher water evaporation, greater vegetation water stress, photosynthetic suppression, and increased plant mortality (McDowell and Allen 2015; Liu et al. 2021; Yan et al. 2025); others indicate that higher temperatures can also lead to improved photosynthetic rates or extended growing seasons (Zhu et al. 2016), provided water availability is not a limiting factor. Therefore, under climate change scenarios, examining the impacts of temperature on NDVI patterns can shed light on large-scale ecosystem changes (de Jong et al. 2011; Liu et al. 2019). As for annual average rainfall, our findings do not support previous long-term studies that showed significant increase in rainfall levels over time in the UK (Kendon et al. 2024). This may be due to the shorter timeframe considered in this study, which might not be sufficiently sensitive to detect significant changes in rainfall trends. Additionally, this difference may be attributed to regional rainfall patterns that deviate from national trends. Nonetheless, studies show that intra-annual rainfall patterns are shifting, with more concentrated (over time) and intense (higher volume) rainfall events that can lead to floods, followed by droughts (Kendon et al. 2024; Kew et al. 2024). We would expect these shifting rainfall patterns to increase water stress, leading to declining trends in NDVI (Zhao and Running 2010; Liu et al. 2025). Contrastingly, shifts may be compensated by longer growth periods due to higher average temperatures, leading to an overall upward trend in NDVI (Zhu et al. 2016). The role of climate variables in the spatio-temporal patterns of ecosystem functioning spectral proxies should be explored in future studies, for instance, by assessing the impacts of intra-annual variation (seasonal trends) and extreme weather events on spectral indices at the landscape level.

While we found a marginally significant correlation between average and minimum temperatures and annual average NDVI over time, the overall increase in NDVI might be influenced by other interacting drivers at the landscape level. Some studies have reported an increase in NDVI over the past decades, primarily attributed to the carbon dioxide fertilization effect, nitrogen deposition, climate change, and LULCC (Zhu et al. 2016; Piao et al. 2020; Liu et al. 2021; Li et al. 2024). Nonetheless, these patterns are not globally homogeneous, and the influence of different drivers may vary at various spatial and temporal scales (Liu and Wennberg 2024). In fact, in our study area, NDVI trends show spatially heterogeneous patterns despite the overall increase. This is corroborated by the spatial autocorrelation distribution patterns, which showed both random and significant clustered spatial distributions. Overall, these results demonstrate that NDVI change patterns exhibit both a widespread moderate random increase and hotspots of NDVI change, with either steep negative or positive trends, indicating the influence of different drivers at various scales. These findings emphasise the importance of understanding ecosystem functioning trends at scales relevant to socio-ecological systems by analysing the drivers of change that shape landscape patterns.

LCC have been linked to shifting NDVI patterns with contrasting effects (Xue et al. 2021). Negative impacts on NDVI are associated with habitat loss and fragmentation, whereas restoration efforts and land abandonment processes tend to trigger natural regeneration and vegetation greenness (Zhu et al. 2016; Krause et al. 2022; Elphick et al. 2024). Our results align with the influence of LCC on NDVI patterns, showing significant differences in MK *tau* values in many areas where LCC were detected. Strong increases and decreases in MK *tau* values associated with LCC (*e.g.* transition from arable to coniferous woodland and grassland to arable, respectively; Fig. 7), indicate that LCC partly explain the steeper NDVI increases (High-High) and decreases (Low-Low) in spatially autocorrelated areas. LCC typically associated with intensification processes and habitat loss show reduced MK values in relation to stable land cover types. Conversely, LCC linked to restoration and conservation initiatives, such as the conversion of grasslands and arable fields to woodlands or natural regeneration sites (Appendix 4), consistently show higher greening trends when compared to stable land cover types. These findings highlight how NDVI trends are closely linked to different LCC directions. However, our LCC analysis do not fully capture all the changes that occurred during the study period, due either to limitations of the land cover maps used (*e.g.* spatial resolution and misclassifications) or the dynamics of land management practices (*e.g.* a common farming practice of crop rotation to improve soil health can lead to an overestimation of LCC in arable land). Our results indicate that the landscape scale analysis captures the cumulative effects of local changes (*e.g.* LULCC) while also providing a critical link to broader NDVI patterns, thereby reflecting how land management decisions shape ecosystem distribution, productivity and dynamics. Future studies should consider additional factors influencing NDVI by integrating complementary datasets, thereby improving our understanding of ecosystem functioning across space and through time, such as LiDAR-derived products to measure canopy cover and vegetation structural complexity. Such higher resolution landscape-level datasets help elucidate the mechanistic links between vegetation structure and broader-scale ecosystem functioning patterns, and their relationships with drivers of change (Glenn et al. 2008). Finally, drivers beyond climatic and ecological factors, such as policy (*e.g.* agricultural, forestry, and nature protection policies) and economic drivers (*e.g*. land tenure, consumer demands, market price fluctuations, and subsidies), may mitigate or intensity the impacts of drivers of change (Hersperger and Bürgi 2009). Understanding these interactions is essential for informing landscape management changes in response to environmental and socio-political challenges.

Overall, this study uncovers the spatio-temporal patterns of NDVI change in landscapes in the South of UK. Analogous trends to those observed in Sussex might be observed in locations with comparable climate and vegetation patterns, and where similar increases in average temperature have been detected. Nonetheless, these locations may show dissimilar trends due to the overlaying effects of other drivers, such as LULCC (Xue et al. 2021; Elphick et al. 2024). As global greening trends continue, especially coupled with climate change, cascading effects on ecosystem functioning are expected to occur in the future, such as increased respiration rates leading to reduced carbon uptake and increased water stress, which will influence NDVI differently from current trends (Liu et al. 2021; Curran and Curran 2025; Zhang et al. 2025). While local and global studies have examined the mechanistic processes driving NDVI changes, integrating ecosystem monitoring at the landscape scale is crucial for advancing landscape approaches capable of connecting the impact of climate changes, global restoration goals, and local management actions. Finally, this study highlights the importance of landscape-scale research, as the landscape is both a scale at which environmental change is perceived and at which science can be translated into action by fostering land management synergies amongst stakeholders and enabling more effective responses to anthropogenic global changes that consider the socio-cultural dynamics and values.

## Conclusions

A lack of spatial and temporal evidence of changes in ecosystem functioning at the landscape level hinders the ability to design and implement effective restoration plans. Our study demonstrates how remote sensing technology can be used to monitor long-term trends in ecosystem functioning and to evaluate the impacts of climate change and land cover changes at the regional and landscape levels. Our study reveals that the climate in Sussex has become warmer over the past three decades, and this warming trend correlates with increased NDVI values. We provide spatially explicit data on the temporal patterns of NDVI change and evidence that temperature-related variables influence NDVI at broader scales, while LCC affect NDVI at a local scale. As landscapes face increasing pressures from multiple drivers at different spatial and temporal scales, ecosystem monitoring at the landscape level is fundamental for shedding light on ecological trends and informing land management and restoration plans.

## Supplementary Information

Below is the link to the electronic supplementary material.Supplementary file1 (DOCX 1173 KB)

## Data Availability

The data and scripts that support the findings of this study are available at https://zenodo.org/records/15488180 and https://github.com/guivcastro/NDVI-timeseries, respectively.
