# Supplementary material for: Uncovering the spatio-temporal patterns and drivers of 30 years of greening in South UK landscapes
Source: Landsc Ecol. 2026 May 25;41(8):129. doi: 10.1007/s10980-026-02383-2 (PMC13385007; doi:10.1007/s10980-026-02383-2)

*Supplementary materials*

**Appendix 1.** Global Moran’s I values obtained for increasing K-nearest neighbour K values.

| \| K \| Global Moran's I \| p-value \| z-score \| \| --- \| --- \| --- \| --- \| \| 2 \| 0.91 \| 0.00 \| 2187.2 \| \| 3 \| 0.89 \| 0.00 \| 2856.7 \| \| 4 \| 0.88 \| 0.00 \| 3005.9 \| \| 5 \| 0.87 \| 0.00 \| 3470.1 \| \| 6 \| 0.86 \| 0.00 \| 3881.6 \| \| 7 \| 0.85 \| 0.00 \| 3959.6 \| \| 8 \| 0.84 \| 0.00 \| 4070.5 \| \| 9 \| 0.84 \| 0.00 \| 4311.8 \| \| 10 \| 0.83 \| 0.00 \| 4497.2 \| \| 11 \| 0.82 \| 0.00 \| 4762.2 \| \| 12 \| 0.82 \| 0.00 \| 5010.1 \| \| 13 \| 0.81 \| 0.00 \| 5068.7 \| \| 14 \| 0.80 \| 0.00 \| 5139.0 \| \| 15 \| 0.80 \| 0.00 \| 5289.5 \| \| 16 \| 0.79 \| 0.00 \| 5401.0 \| \| 30 \| 0.72 \| 0.00 \| 6791.5 \| \| 50 \| 0.66 \| 0.00 \| 8073.0 \| \| 100 \| 0.57 \| 0.00 \| 9833.4 \| |
| --- | --- | --- | --- | --- | --- | --- | --- | --- | --- | --- | --- | --- | --- | --- | --- | --- | --- | --- | --- | --- | --- | --- | --- | --- | --- | --- | --- | --- | --- | --- | --- | --- | --- | --- | --- | --- | --- | --- | --- | --- | --- | --- | --- | --- | --- | --- | --- | --- | --- | --- | --- | --- | --- | --- | --- | --- | --- | --- | --- | --- | --- | --- | --- | --- | --- | --- | --- | --- | --- | --- | --- | --- | --- | --- | --- | --- |

**Appendix 2***.* Distribution of Mann-Kendall *tau* values.


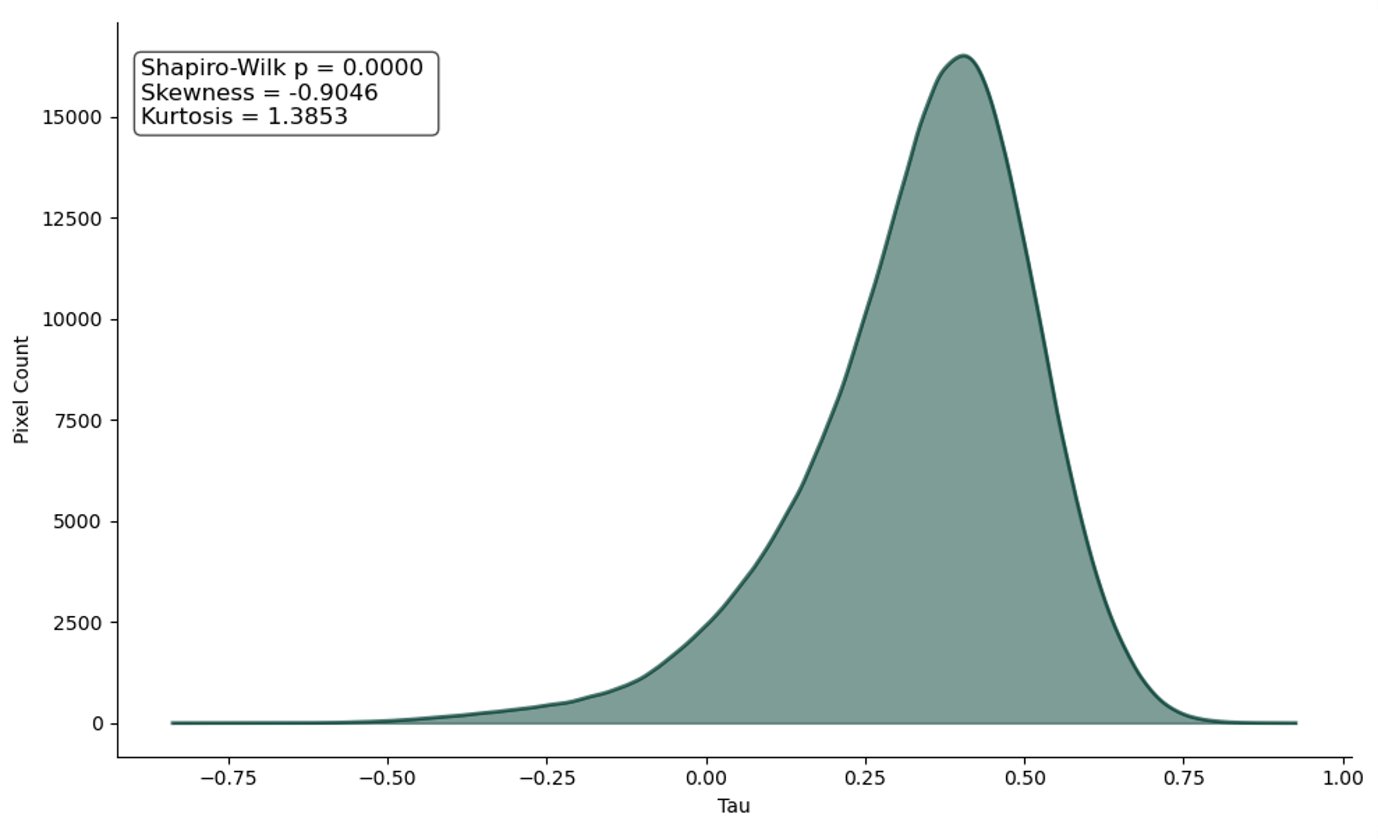


**Appendix 3.** Graphs showing the distribution of Local Moran’s I values for the entire study area and for each land cover type with full x-axis range values.


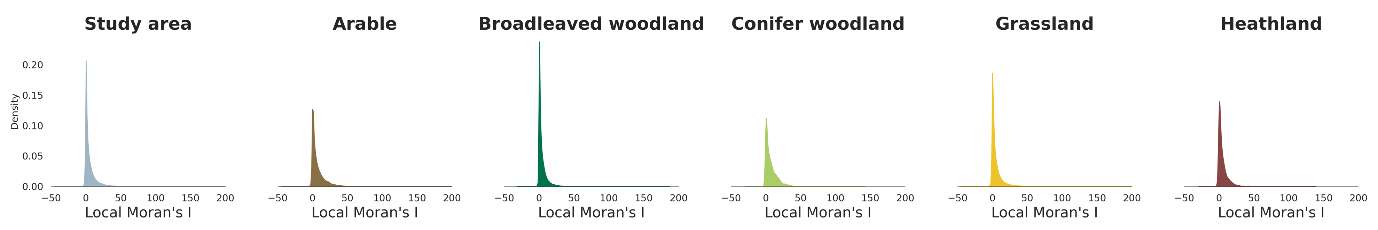


**Appendix 4***.* Mann-Kendall NDVI trend analysis from 1995 to 2024. A – An example of a strong negative trend in a grassland area transformed into an arable farmland; B – An example of a strong positive trend as a result of the rewilding of arable farmland. Pixels where NDVI increases with time are represented in blueish colour (positive MK *tau* values), pixels where NDVI decreases with time are coloured in brown (negative MK *tau* values), and pixels showing no significant NDVI trends are coloured in grey.


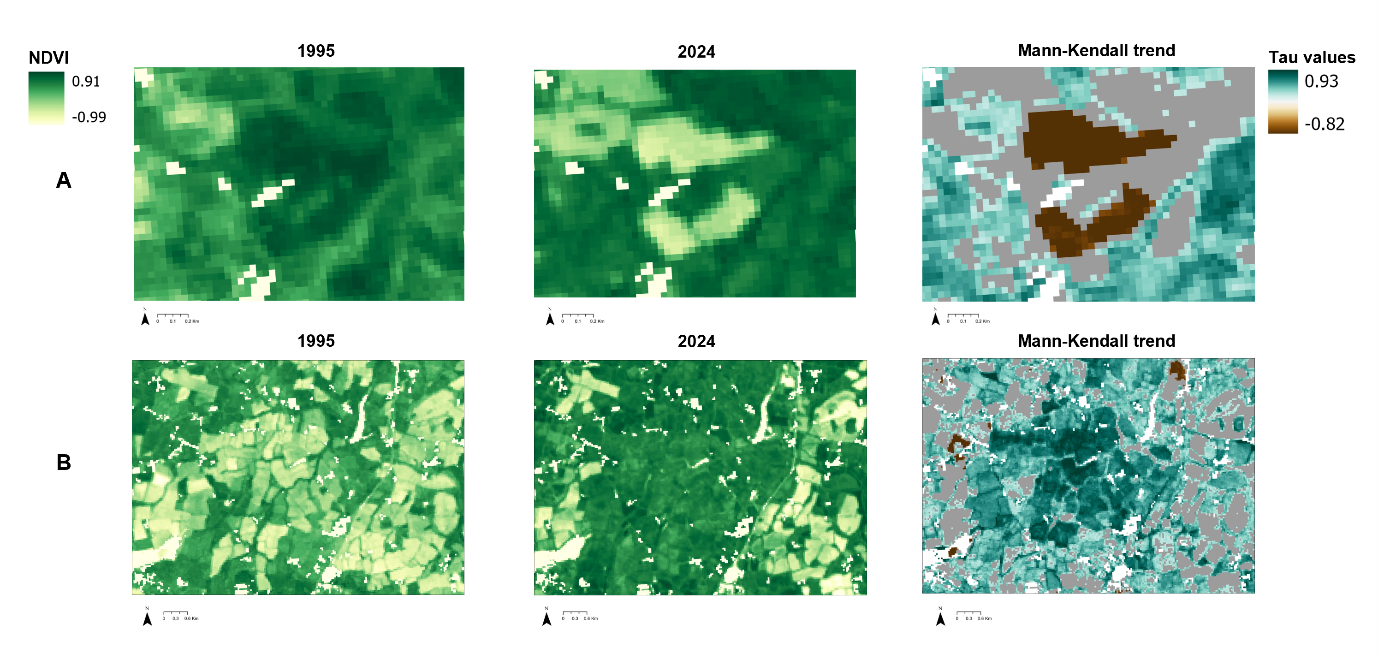

Supplement: Supplementary file 1 — Supplementary file1 (DOCX 1173 KB) [file 10980_2026_2383_MOESM1_ESM.docx]
